# Utility of extracorporeal membrane oxygenation (ECMO) in the management of traumatic tracheobronchial injuries: case series

**DOI:** 10.1093/jscr/rjab158

**Published:** 2021-04-24

**Authors:** Hassan Al-Thani, Khalid Ahmed, Sandro Rizoli, Talat Chughtai, Ibrahim Fawzy, Ayman El-Menyar

**Affiliations:** 1 Trauma Surgery Section, Department of Surgery, Hamad General Hospital (HGH), Doha, Qatar; 2 Medical Intensive Care Unit, HGH, Doha, Qatar; 3 Clinical Research, Trauma and Vascular Surgery Section, HGH, Doha, Qatar; 4 Clinical Medicine, Weill Cornell Medical College, Doha, Qatar

## Abstract

Tracheobronchial injury is a rare, but potentially life-threatening condition, and in most cases requires urgent treatment to restore normal respiratory physiology. Over the past decades, extracorporeal membrane oxygenation (ECMO) has evolved as an important adjunct in airway surgery. We presented three cases of traumatic tracheobronchial injury managed with ECMO support at a level-1 trauma center and emphasized the benefits of anticipation and early institution of ECMO support perioperatively, in these high-risk cases. The management of traumatic tracheobronchial injuries requires early measures to guarantee adequate ventilation. Anticipation and early institution of ECMO in these patients may support respiratory physiology, facilitate repair and improve survival. The time factor and multidisciplinary communication and plan prior to intervention should be considered. ECMO support, whenever available, plays important role in the management of complicated tracheobronchial surgical procedure and thereby reduces risk of mortality.

## INTRODUCTION

Tracheobronchial injury after blunt trauma is a rare, but life-threatening requiring urgent treatment (including surgical repair) in most cases to restore normal respiratory physiology [1]. Despite the large increase in trauma cases in recent years, tracheobronchial injuries remain uncommon and not easy to be early diagnosed [2]. They are usually seen in cases of severe polytrauma, but may go unrecognized, and thus untreated, due to difficulty in diagnosis, as the presenting signs and symptoms often do not correlate with the severity of the injury. Injuries may range from a simple endobronchial hematoma, to a complex or complete tracheal transaction [3]. The presenting symptoms and signs often do not reflect the severity of the injury, and therefore a high index of suspicion needs to be maintained depending on the mechanism of injury. Diagnosis may be made clinically, bronchoscopically, radiologically or intraoperatively. Treatment may include observation with supportive care, endoluminal stents, primary repair, as well as reconstruction of the airway [3]. Over the past decades, extracorporeal membrane oxygenation (ECMO) has evolved as an important adjunct in airway surgery, and in cases of ineffective oxygenation/ventilation, ECMO support may be used as a salvage therapy perioperatively [4–5]. Evidence is now emerging that early implementation of ECMO may limit, or even reverse, the extent of multisystem organ failure resulting from trauma-related sequelae, traditionally associated with high mortality, especially in the setting of severe chest injuries [6]. Typically, ECMO is indicated in the setting of severe hypoxemia and/or hypercarbia, which are associated with a mortality in excess of 80% using conventional ventilation strategies [7]. In terms of overall utility of ECMO, the ‘Conventional Ventilation or ECMO for Severe Acute Respiratory Failure’ (CESAR) trial showed that patients referred to an ECMO center had a significant increase in survival without disability at 6 months compared to conventional management (63% versus 47%, respectively) [8]. Herein, we present three cases of traumatic tracheobronchial injury managed with ECMO support at a level-1 trauma center, to emphasize the benefits of anticipation and early institution of ECMO support in these high-risk cases.

## CASE SERIES

### Case 1—bronchial injury

A 27-year-old male presented to the Trauma Room after a fall from a 3-meter height. Because of low Glasgow coma scale (GCS), hypoxia and diffuse subcutaneous emphysema, the patient was intubated, and bilateral chest tubes were inserted. After stabilization, a pan computed tomography (CT) scan was performed and revealed extensive subcutaneous emphysema of the neck and chest, as well as bilateral pulmonary contusion. The patient was admitted to the Trauma intensive care unit (ICU) for supportive treatment, where he continued to have hypoxia as well as a persistent right-sided pneumothorax on a repeated chest X-ray. Because a major airway injury was suspected, a bronchoscopy was performed, which revealed a full-thickness laceration in the region of the bronchus intermedius/right middle lobe bronchus. Due to persistent hypoxia, the ECMO team was consulted and the patient was started on Veno-Venous (VV) ECMO. With ECMO support, he was shifted to the operating theater (OT) for right anterolateral thoracotomy and repair of the bronchial defect. The surgery was uneventful, and the patient returned to the ICU in stable condition. He remained sedated, on lung-protective ventilation, as well as ECMO support. The patient underwent tracheostomy on Day 5 and was successfully removed from ECMO and decannulated on Day 16. He remained in the ICU for 25 days, after which he was transferred to the ward in stable condition. Follow-up flexible bronchoscopy showed an intact bronchial tree, with only a partial occlusion of the lumen. The patient was discharged home in stable condition with regular follow-up in the outpatient clinic.

**Figure 1:**
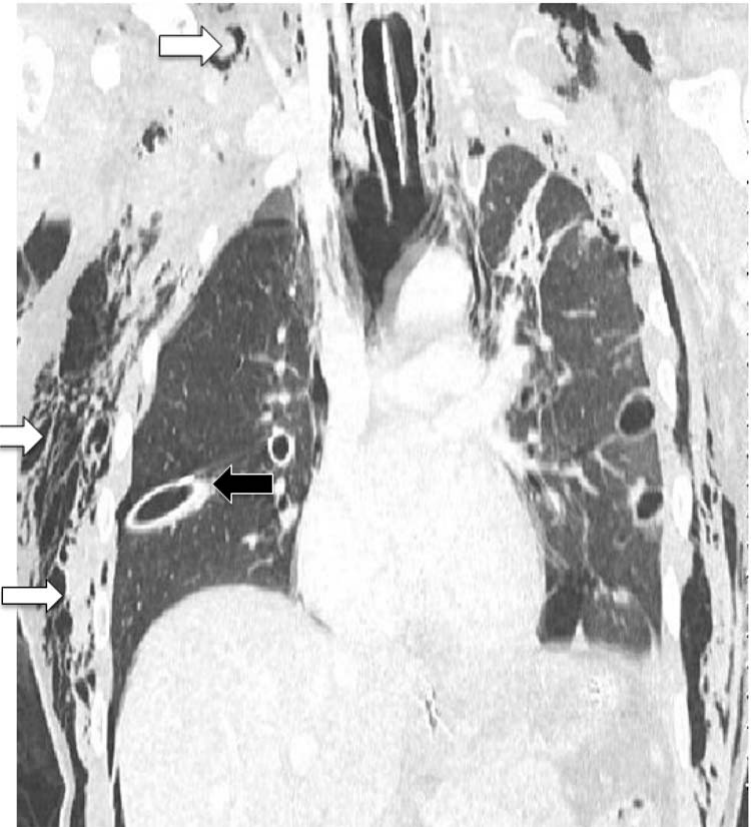
CT chest showing chest tubes in situ (black arrow) and soft tissue emphysema (white arrows).

### Case 2—bronchopleural fistula

A 63-year-old male was brought to the Trauma Room after a fall from height. On initial assessment, he was hypotensive and hypoxic, with decreased air entry bilaterally, as well as significant subcutaneous emphysema. Bilateral chest tubes were inserted, and the patient was intubated, after which his saturation decreased to 60%. The patient was stabilized after insertion of two additional chest tubes and underwent a pan-CT scan, which showed extensive surgical emphysema in the neck and thoracic wall, with extension to the right lower abdominal wall. There were multiple rib fractures and bilateral lung contusion, as well as a right lung laceration with persistent pneumothorax. Because of the presence of pneumoperitoneum, the patient underwent an exploratory laparotomy. Intraoperatively, his O_2_ saturation remained critically low and he was shifted to the ICU on 100% FiO_2_. The ECMO team was consulted for severe hypoxemia (as well as hypercarbia) most likely due to ARDS secondary to severe traumatic pulmonary contusion. Prone ventilation was attempted; however, the patient immediately developed a large air leak from one of the right-sided chest tubes, and the minute ventilation dropped by almost half. The patient was replaced on the supine position, cannulated, and started on VV-ECMO support, for severe refractory hypoxemia caused by a bronchopleural fistula. This fistula was initially missed by the bronchoscopy as it was distally located; however, the diagnosis was mad by the clinical and CT scan findings. The patient was kept on ultraprotective ventilator settings, with full ECMO support for ~2 weeks. Several bronchoscopies were performed for right lung collapse, and no proximal airway injury was identified. On Day 16, due to fever and radiologic evidence of empyema, the patient underwent right Video Assisted Thoracic Surgery (VATS), drainage of almost 1 L of purulent material, and decortication of the lung. He gradually improved, the chest drains were systematically removed, and the patient was successfully weaned off ECMO, and decannulated on Day 28. He underwent percutaneous dilatational tracheostomy (PDT) and remained in ICU for a total of 54 days, after which he was shifted to the ward. On Day 62 post-trauma, he was discharged to the rehabilitation unit.

### Case 3—tracheal injury

A 29-year-old male presented to the Trauma Unit, after being hit by a car. Because of desaturation, diminished air entry bilaterally and subcutaneous emphysema, he was intubated, and bilateral chest tubes were placed. After stabilization, a pan-CT scan revealed extensive soft tissue emphysema in the neck and chest, as well as pneumomediastinum. An additional two chest tubes were placed bilaterally because of incomplete lung expansion (Fig. 1). The patient improved over several days and was eventually discharged home in stable condition.

**Figure 2:**
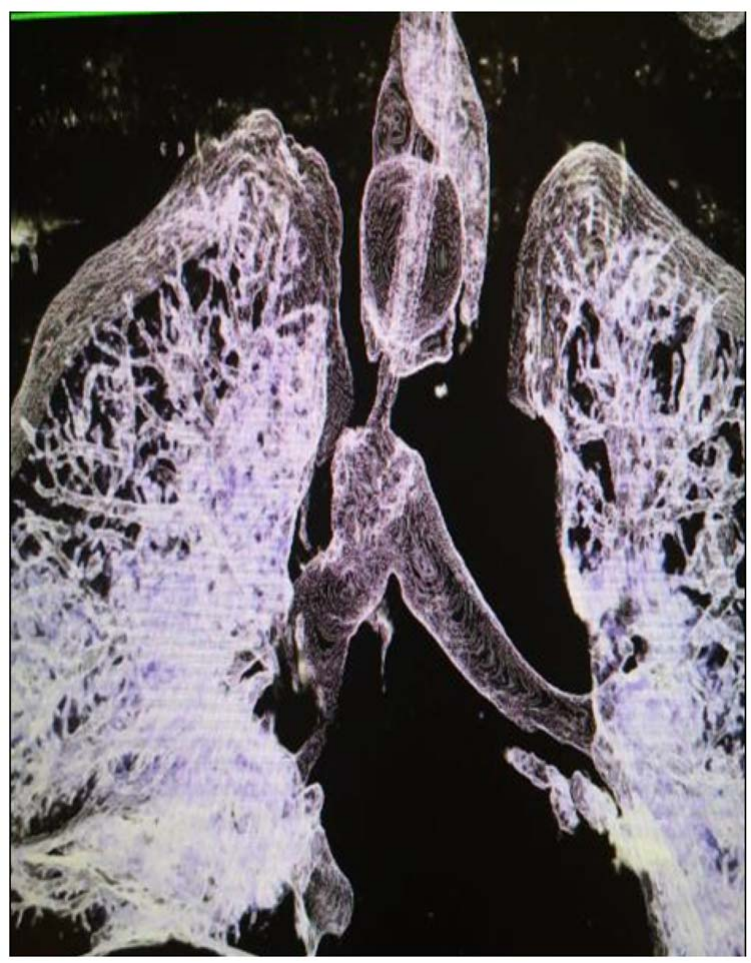
Reconstructed CT scan showing injury site of trachea.

**Figure 3:**
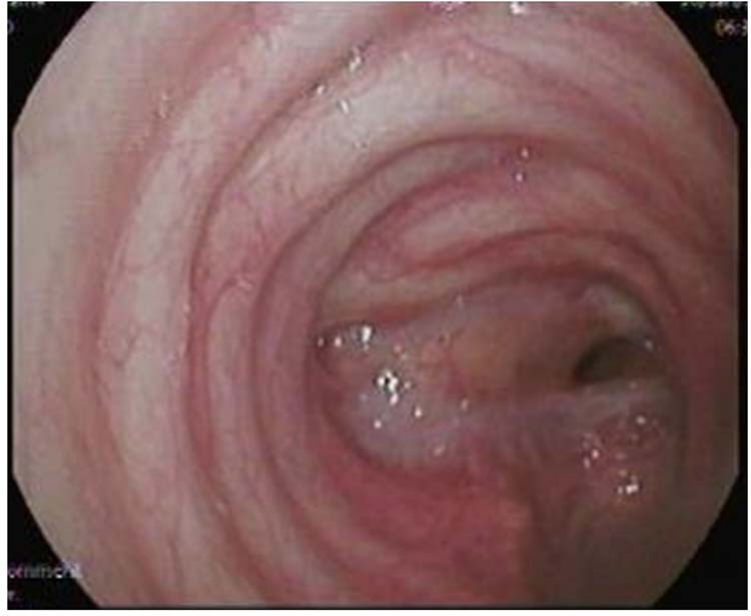
Flexible bronchoscopy showing tight tracheal narrowing.

**Figure 4:**
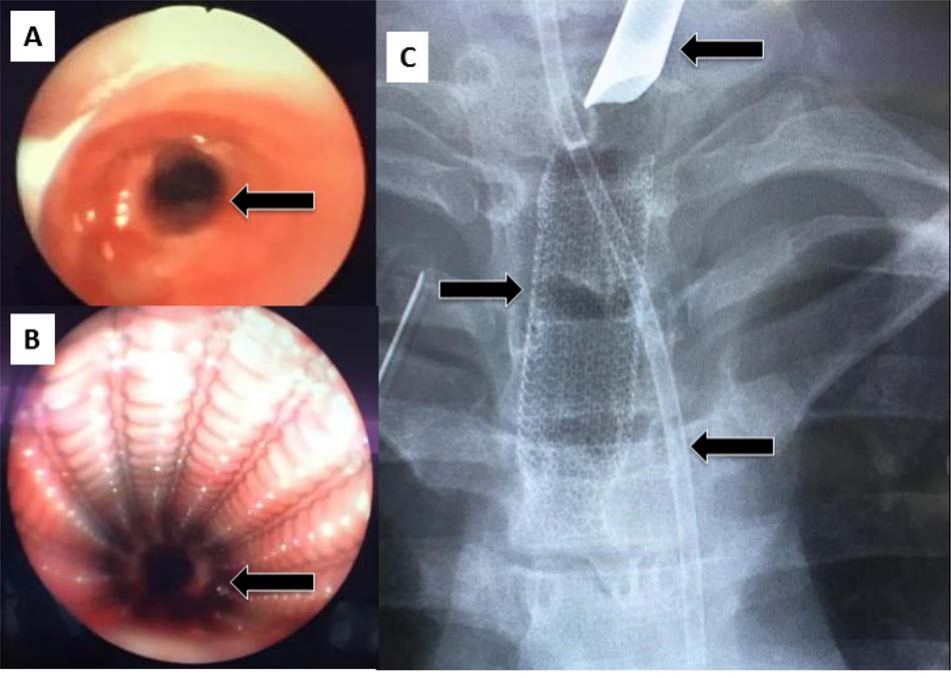
(**A**) Rigid bronchoscopy show 4 mm stenosis of the distal trachea, (**B**) intra-tracheal showing the stent following dilatation and placement of stent, (**C**) plain X-ray showing stent in place, feeding tube and endotracheal tube.

After discharge, the patient presented to the Emergency Room with shortness of breath and tachypnea. Chest X-rays and CT scan were unremarkable; however, he was admitted with type 2 respiratory failure and placed on noninvasive ventilation. Due to persistent tachypnea, the patient’s initial CT scans were reassessed with reconstruction and showed a complete transection of the trachea that was overlooked initially (Fig. 2). Flexible bronchoscopy revealed a significant post-traumatic tracheal stenosis (Fig. 3). It was tight stenosis (2 cm length on the CT reconstruction) and just above the carina. The patient was taken to the OT, with the ECMO team on standby. During rigid bronchoscopy, the patient became more hypoxic, and VV-ECMO was instituted. The stenosis was dilated with a small (#6) endotracheal tube (ETT) placed across the stenosis. He subsequently returned to the OT for further dilation and placement of a # 7.5 ETT. After 9 days, bronchoscopy revealed no gross residual stenosis and therefore the patient was successfully extubated. The patient was removed from ECMO support on the following day. After several days, the patient developed tachypnea, tachycardia and a respiratory acidosis. He was reintubated and shifted to the OT again (with ECMO standby) for redilation of restenosis with a #8 ETT in place. A covered tracheal stent was placed intraoperatively via rigid bronchoscopy (Fig. 4  **A–C**). Later on, the patient was extubated and eventually discharged home in stable condition.

**Figure 5:**
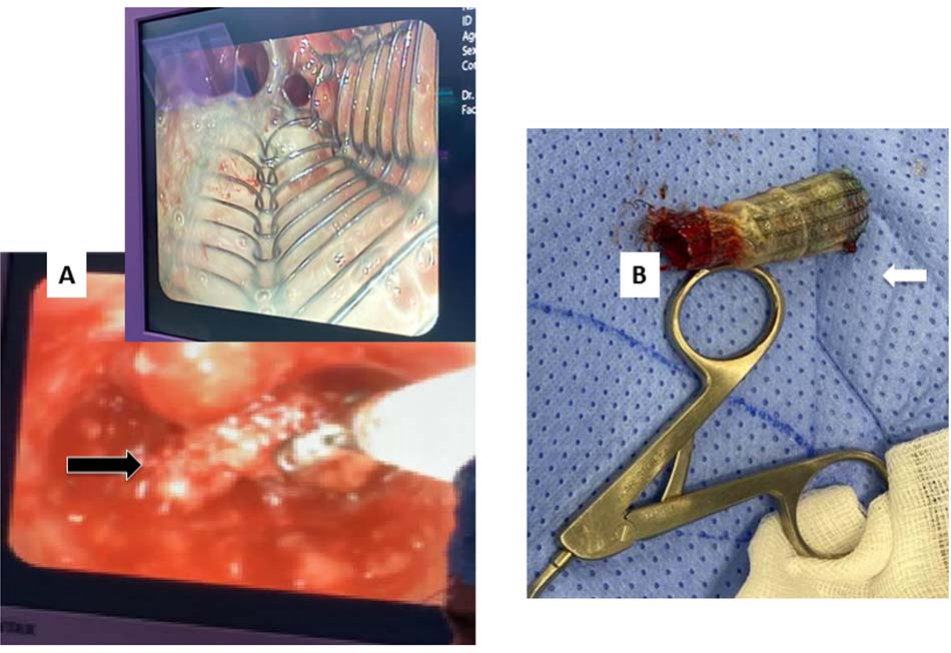
(**A**) Rigid bronchoscopy revealed the lower end of the stent to be embedded in granulation tissue. (**B**) The stent after removal showing the distal part with granulation tissue.

After 1 month, the patient again presented to the ED with stridor and respiratory distress and was shifted to the OT. Bronchoscopy revealed granulation tissue obstructing the distal tracheal margin. A 7.5 sized ETT was passed through the stenotic site, with a plan for removal of the granulation tissue and replacement with a new (longer) stent. Rigid bronchoscopy revealed the lower end of the stent to be embedded in granulation tissue. Blunt dissection was performed to free up and removal of the stent (Fig. 5A and B). However, immediately after stent removal, the patient developed severe hypoxia, hypotension and bradycardia. The cervical trachea was opened longitudinally, and attempts were made to pass the ETT tube ‘manually’ across the stenosis, which also failed. At this point, the patient went into cardiac arrest, and CPR was started. A right anterolateral thoracotomy and lower sternotomy were performed, with open cardiac massage. The mediastinal trachea was exposed and opened anteriorly/inferiorly, and two small ETT’s were placed into each main bronchus. Attempts to establish an opened airway distal to the stenosis and to resect it was not successful as the patient condition quickly and progressively deteriorated. Unfortunately, despite resuscitative efforts, the patient was declared dead.

## DISCUSSION

Optimal management of tracheobronchial injuries necessitates a multidisciplinary team including trauma surgeon, intensivist, anesthesiologist and cardiothoracic surgeon in a highly specialized center. The clinical recognition of tracheabronchial injury requires a high index of suspicion, imaging and appropriate urgent intervention. Successful surgical repair demands good exposure of the injury and effective control of ventilation and oxygenation perioperatively [5]. Over the past decades, with the evolution of its circuits and centrifugal pumps, ECMO has become popular for thoracic surgery, including trauma, in order to avoid the inherent risks and complexity of cardiopulmonary bypass [4]. Specifically, in cases of failing and ineffective ventilation, ECMO support may be considered a very useful tool for efficient gas exchange during surgery for traumatic tracheobronchial injury [4–5].

In our first case, there was clinical suspicious of a bronchial injury and thus ECMO was rapidly instituted. Early institution of ECMO in such injury has been shown to improve survival as a bridge for the definite intervention [9–10]. Walker *et al.,* provided the first description of cardiopulmonary support for a tracheobronchial injury in an adult patient. He described successful surgical repair of a transected right main bronchus utilizing VV- ECMO [10].

In the second case of this series, the clinical findings raised suspicion of an airway injury. When prone positioning was attempted and a large air leak was noted from one of the chest tubes with a significant drop of the minute ventilation, the patient was then started on VV-ECMO. The severe respiratory failure was secondary to lung contusion, pneumothorax and bronchopleural fistula. Despite the complication of empyema, the patient was kept on ECMO support until his condition improved, and successfully weaned from ECMO. Rinieri *et al.* [10], similarly described the successful use of ECMO in a patient with bronchial fistula with a favorable outcome.

ECMO can be an efficient backup or salvage option in managing multisystem trauma patients with tracheobronchial injuries. Complete blunt traumatic tracheal transection is extremely rare and life-threatening that requires urgent repair to restore ventilation [3]. Enomoto *et al.,* described the use of perioperative VV- ECMO in a patient with a traumatic tracheal transection 10 mm above the tracheal bifurcation [11]. Hong *et al.* [12], described successful tracheal stent removal, on ECMO support. Advantages with the use of ECMO during tracheal surgery include the possibility of avoiding the use of cross-field ventilation with endotracheal cuffed tubes or high-frequency jet ventilation catheters, improving visualization of the surgical field and avoiding intermittent ventilation that may be required during surgery [10]. Carretta *et al.* [1] described the successful use of ECMO and end-to-end tracheal anastomosis in a young man, involved in a motor vehicle collision. In the third case of the present series, unfortunate events occurred including missed and underestimated lesion ultimately led to succumb of the patient. The patient was referred to thoracic surgery and was in poor conditions to undergo surgery and requiring urgent intervention. Following the review of the CT scan, complete disruption of the trachea with nearly 2.5 cm length of tight stenosis in the lower third of the trachea with a short distal segment of trachea above the carina was found. Based on the CT findings and the intraoperative bronchoscopy, a partially covered metallic stent was used. Following dilatation using flexible bronchoscope, the delivery of the stent was made. This self-expanding stent allows restoration of the tracheal diameter and has good proximal and distal adherence to the wall of the trachea to prevent the migration risk. Silicone stent placement was not considered due to the lack of appropriate size at the time of the procedure. During the intervention for granulation tissue removal and stent replacement, ECMO support was not available as all the ECMO machines were already in use for COVID-19 patients. Attempts to deal with such situation were extremely challenging and eventually failed. The time factor, multidisciplinary communication and plan prior to replacement of the stent should be considered.

## CONCLUSIONS

Traumatic tracheobronchial injuries are uncommon and life-threatening conditions that require early measures to guarantee adequate ventilation. The time factor and multidisciplinary communication and plan prior to intervention should be considered. ECMO support, whenever available, plays important role in the management of complicated tracheobronchial surgical procedure and thereby reduces risk of mortality.
